# PD-L1 inhibitor versus PD-1 inhibitor plus bevacizumab with transvascular intervention in unresectable hepatocellular carcinoma

**DOI:** 10.1007/s10238-024-01415-y

**Published:** 2024-06-28

**Authors:** Zhenkun Huang, Tiejun Chen, Wenbin Li, Jiliang Qiu, Shaoru Liu, Zongfeng Wu, Binkui Li, Yunfei Yuan, Wei He

**Affiliations:** 1 State Key Laboratory of Oncology in South China, Guangdong Provincial Clinical Research Center for Cancer, Sun Yat-Sen University Cancer Center, Guangzhou, China; 2 Department of Liver Surgery, Sun Yat-Sen University Cancer Center, Guangzhou, China; 3 Department of Hepatobiliary Surgery, The Affiliated Cancer Hospital & Institute of Guangzhou Medical University, Guangzhou, China; 4 Department of Biliopancreatic Surgery, Sun Yat-Sen Memorial Hospital, Guangzhou, China

**Keywords:** PD-L1 inhibitors, PD-1 inhibitors, Unresectable hepatocellular carcinoma

## Abstract

Both atezolizumab (a PD-L1 inhibitor) plus bevacizumab (A+B) and sintilimab (a PD-1 inhibitor) plus bevacizumab (S+B) are recommended as the first-line regimen for advanced hepatocellular carcinoma (HCC) in China. Different efficacy between the two regimens combined with transvascular intervention for unresectable HCC (uHCC) remain unknown. We retrospectively analyzed uHCC patients treated in three centers by simultaneous combination of A+B or S+B with transarterial chemoembolization (TACE) and FOLFOX-based hepatic arterial infusion chemotherapy (HAIC). Objective response rate (ORR), progression-free survival (PFS), overall survival (OS) and treatment-related adverse events (TRAEs) were compared. Totally 188 patients were included, with 92 and 96 administered A+B+TACE-HAIC (ABTH) and S+B+TACE-HAIC (SBTH), respectively. ORRs (62.0 vs. 70.8%, respectively; *P* = 0.257) and disease control rates (88.0 vs. 93.8%, *P* = 0.267) were similar between groups by the mRECIST criteria. ABTH showed no survival advantage over SBTH, with median PFS times of 11.7 months and 13.0 months, respectively (HR = 0.81, 95% CI, 0.52–1.26, *P* = 0.35) and similar OS times (HR = 1.19, 95% CI, 0.32–4.39, *P* = 0.8). No significant differences were observed in grade 3–4 TRAEs between groups. Either PD-L1 or PD-1 inhibitor plus bevacizumab combined with TACE-HAIC have similarly excellent therapeutic efficacy with manageable adverse events, representing promising treatment options for uHCC.

**Supplementary Information:**

The online version contains supplementary material available at 10.1007/s10238-024-01415-y.

## Introduction

Immune checkpoint inhibitors are an important part of systemic therapy for advanced hepatocellular carcinoma and provide palliative care for patients [1–5]. The PD-L1 inhibitor, atezolizumab, combined with bevacizumab (A+B) significantly improved response rate (ORR) (33.2% vs. 13.3%), overall survival (1-year OS, 67.2% vs. 54.6%) and progression-free survival (mPFS; 6.8 months vs. 4.3 months) compared to sorafenib alone in the IMBrave150 trial, rendering A+B as the first-choice for the treatment of advanced HCC [6, 7]. However, a PD-1 inhibitor, pembrolizumab, in combination with lenvatinib did not significantly improve survival in patients with advanced HCC compared to lenvatinib alone in the LEAP-002 trial [8]. The results of the two above phase III studies, one successful but the other failed, suggest that whether there is a difference in the efficacy of PD-1 or PD-L1 inhibitor combined with targeted agents in advanced HCC deserves further exploration. The ORIENT-32 trial performed in China reported that another PD-1 inhibitor, sintilimab, in combination with a bevacizumab biosimilar (S+B) significantly improved efficacy (ORR: 24% vs. 8%, median OS not reached vs. 10.4 months, median PFS 4.6 months vs. 2.8 months) compared to sorafenib monotherapy in advanced HCC [9]. Based on the positive results of the IMbrave150 and ORIENT-32 trials, both A+B and S+B regimens have been approved as the first-line treatment for advanced HCC in China. The two trials were very similar in design, but examined distinct target populations. In addition, atezolizumab and sintilimab are PD-L1 and PD-1 antibodies, respectively, and it is worth exploring whether there is a difference in efficacy between PD-L1 and PD-1 inhibitors both in combination with bevacizumab in the same population. Because a prospective head-to-head study is not feasible, the significance of the present real-world study could bridge the existing gap.

Previous studies by our team have demonstrated the high efficacy of a novel treatment approach combining transarterial chemoembolization (TACE) and hepatic arterial infusion chemotherapy (HAIC) in patients with potentially resectable HCC or portal vein tumor thrombus (PVTT) [10, 11]. However, it is unknown whether the combination of immune checkpoint inhibitors (ICIs) and macromolecular VEGF-targeted therapy with transvascular local interventions could improve patient prognosis in uHCC. Moreover, whether targeting distinct immune checkpoints (PD-1 vs. PD-L1) results in different efficacy levels for locoregional therapy remains undefined. The aim of this study was to evaluate and compare the efficacy and safety of combining A+B or S+B with TACE-HAIC in patients with uHCC.

## Methods

### Patients and study design

This retrospective real-world study enrolled patients treated in three medical centers in China, including Sun Yat-sen University Cancer Center, Affiliated Cancer Hospital & Institute of Guangzhou Medical University, and Sun Yat-Sen Memorial Hospital. These patients, diagnosed with treatment-naive uHCC, underwent simultaneous combination TACE-HAIC and A+B or S+B between March 2021 and July 2023. Inclusion criteria were: (a) a confirmed diagnosis of uHCC; (b) at least one target lesion evaluable by both RECIST 1.1 and mRECIST criteria; (c) Child-Pugh Grade A or B. Exclusion criteria were: (a) previous exposure to other anti-cancer treatments; (b) diagnosis of any other primary malignancy; (c) significant esophageal varices or observable red wale marks; (d) a history of severe cardiac, pulmonary, or renal comorbidities; (e) incomplete follow-up records. This study is closed to enrolment and is registered with ClinicalTrials.gov (NCT06199297).

### Treatment procedure

The methodology used for TACE-HAIC has been outlined in our prior reports [10, 11]. In essence, the chemoembolization process employed 30 mg/m^2^ of epirubicin and 2–10 mL of lipiodol to reduce the blood flow, but not to completely block the vessels. This was followed by FOLFOX-based HAIC, including 85 mg/m^2^ of oxaliplatin, 400 mg/m^2^ of leucovorin, and an initial bolus of 400 mg/m^2^ of 5-FU for 2 h, which was then followed by a sustained infusion of 1200 mg/m^2^ 5-FU for 23 h, which can improve the efficacy combined with TACE. On the next day, atezolizumab was administered intravenously at 1,200 mg or sintilimab at 200 mg. Bevacizumab was administered intravenously, ensuring a minimum interval of 5 min from the prior drug, at 15 mg/kg. The combined regimens of A+B/S+B with TACE-HAIC were scheduled every 3–4 weeks. Typically, 2–6 cycles of TACE-HAIC were employed; when the endpoint of interventional embolization was reached or the intrahepatic tumor vasculature was unsuitable for vascular intervention, TACE-HAIC was discontinued, and targeted immunotherapy was continued until disease progression or intolerance. After completion of the conversion treatment, the appropriateness for hepatic resection was collaboratively determined by a multidisciplinary consortium comprising surgeons, radiologists, and oncologists.

### Data collection and outcomes

Baseline demographics and follow-up data were extracted from medical records. The parameters collected encompassed gender, age, Eastern Cooperative Oncology Group performance status (ECOG PS) score, etiology, α-fetoprotein (AFP) concentration, platelet count (PLT), albumin (ALB) levels, alanine aminotransferase (ALT) levels, aspartate aminotransferase (AST) levels, total bilirubin (TBIL), prothrombin time (PT), tumor count, predominant tumor dimensions, concurrent liver cirrhosis, tumor thrombosis, extrahepatic metastasis, and incidence rates of treatment-related adverse events (TRAEs).

Tumor responses were classified as progressive disease (PD), stable disease (SD), partial response (PR), or complete response (CR) based on both RECIST 1.1 and mRECIST criteria. The primary outcomes were objective response rate (ORR) and progression-free survival (PFS). The ORR reflected the proportion of patients achieving either a CR or PR. The overall tumor response encapsulated the outcomes of all target lesions, while the intrahepatic target lesion response specifically considered the outcomes of lesions within the liver. PFS denoted the time from the initiation of the combined therapeutic regimen to disease progression or death from any cause. Secondary outcomes encompassed TRAE incidence rates and overall survival (OS), which was the time from the commencement of the combined therapy to death from any cause. The disease control rate (DCR) reflected the proportion of patients achieving a CR, PR, or SD. TRAEs were graded using the Common Terminology Criteria for Adverse Events (CTCAE) v5.0 criteria.

### Statistical analysis

Baseline demographics, tumor responses, and TRAEs were presented as frequency and percentage. Categorical variables among baseline characteristics were compared by the Pearson’s χ^2^ test or Fisher’s exact test. Survival analysis was performed by the Kaplan–Meier method, and differences in survival curves were analyzed by a log-rank test. Treatment (ABTH vs. SBTH) and all variables with *P* < 0.05 in univariate analysis were further assessed in multivariate analysis using Cox regression models. The hazard ratio (HR) and the corresponding confidence interval (CI) were calculated. Two-tailed *P* < 0.05 was considered statistically significant. Statistical analysis was performed with R 4.2.2 or SPSS 26.0.

## Results

### Patient and tumor characteristics

From March 2021 to July 2023, 188 eligible uHCC patients administered A+B/S+B combined with TACE-HAIC treatment were included (Supplementary Fig. S1). Table 1 details the baseline characteristics of these patients. All variables related to treatment efficacy were similar between groups.

**Table 1:** Patient baseline characteristics

Variable, n (%)	ABTH (92)	SBTH (96)	*P*
Age			
< 60	65 ( 70.7)	68 ( 70.8)	1
≥ 60	27 ( 29.3)	28 ( 29.2)	
Gender			
Female	4 ( 4.3)	9 ( 9.4)	0.284
Male	88 ( 95.7)	87 ( 90.6)	
ECOG PS			
0	78 ( 84.8)	85 ( 88.5)	0.067
1	9 ( 9.8)	11 ( 11.5)	
2	5 ( 5.4)	0 ( 0.0)	
Child-pugh grade			
A	88 ( 95.7)	94 ( 97.9)	0.64
B	4 ( 4.3)	2 ( 2.1)	
HBsAg			
Negative	7 ( 7.6)	17 ( 17.7)	0.063
Positive	85 ( 92.4)	79 ( 82.3)	
ALBI			
1	61 ( 66.3)	63 ( 65.6)	1
2	31 ( 33.7)	33 ( 34.4)	
ALT, U/L			
≤ 50	58 ( 63.0)	59 ( 61.5)	0.941
> 50	34 ( 37.0)	37 ( 38.5)	
AST, U/L			
≤ 40	28 ( 30.4)	24 ( 25.0)	0.503
> 40	64 ( 69.6)	72 ( 75.0)	
PLT, 10^9^/L			
> 100	88 ( 95.7)	94 ( 97.9)	0.64
≤ 100	4 ( 4.3)	2 ( 2.1)	
PT,second			
≤ 13.5	78 ( 84.8)	84 ( 87.5)	0.743
> 13.5	14 ( 15.2)	12 ( 12.5)	
AFP, ng/mL			
≤ 400	42 ( 45.7)	40 ( 41.7)	0.686
> 400	50 ( 54.3)	56 ( 58.3)	
BCLC stage			
A	9 ( 9.8)	13 ( 13.5)	0.616
B	16 ( 17.4)	19 ( 19.8)	
C	67 ( 72.8)	64 ( 66.7)	
Tumor number			
Solitary	25 ( 27.2)	23 ( 24.0)	0.735
Multiple	67 ( 72.8)	73 ( 76.0)	
Tumor thrombus			
Absent	29 ( 31.5)	40 ( 41.7)	0.197
Present	63 ( 68.5)	56 ( 58.3)	
Extrahepatic metastasis			
Absent	70 ( 76.1)	76 ( 79.2)	0.74
Present	22 ( 23.9)	20 ( 20.8)	
Liver cirrhosis			
Absent	54 ( 58.7)	55 ( 57.3)	0.962
Present	38 ( 41.3)	41 ( 42.7)	
Main tumor size, cm			
< 10	35 ( 38.0)	42 ( 43.8)	0.518
≥ 10	57 ( 62.0)	54 ( 56.2)	

### Treatment efficacy

The median follow-up times were 9.5 (95% CI, 8.1–11.5) and 9.8 (95% CI, 8.5–12.0) months in the ABTH and SBTH groups, respectively. The median treatment cycles administered were 3 (1–9 cycles) and 3 (1–7 cycles) in the ABTH and SBTH groups, respectively.

**Fig. 1:**
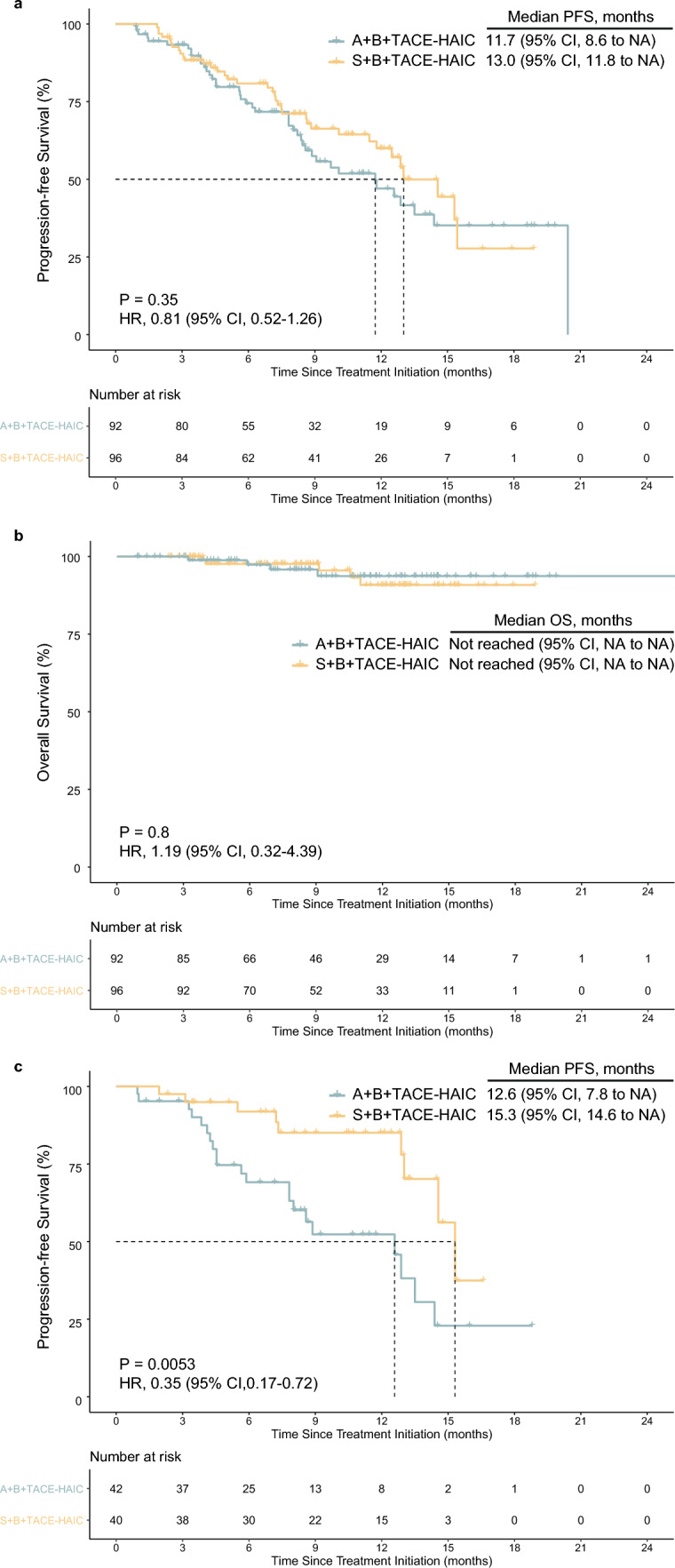
Kaplan–Meier curve for progression-free survival and overall survival.The PFS (**a**) and OS (**b**) for overall population and the PFS (**c**) for patients with AFP ≤ 400 ng/mL. A+B+TACE-HAIC, atezolizumab plus bevacizumab combined TACE-HAIC. S+B+TACE-HAIC, sintilimab plus bevacizumab combined TACE-HAIC

ABTH showed no survival advantage over SBTH, with median PFS of 11.7 months (95% CI, 8.6-NA) and 13.0 months (95% CI, 11.8-NA), respectively (HR = 0.81, 95% CI, 0.52–1.26, *P* = 0.35; Fig. 1a). Median OS data in both groups were still pending (Fig. 1b). One-year OS and PFS rates were 93.7% and 47.0% in the ABTH group, respectively, and 90.8% and 60.0% in the SBTH group, respectively.

**Fig. 2:**
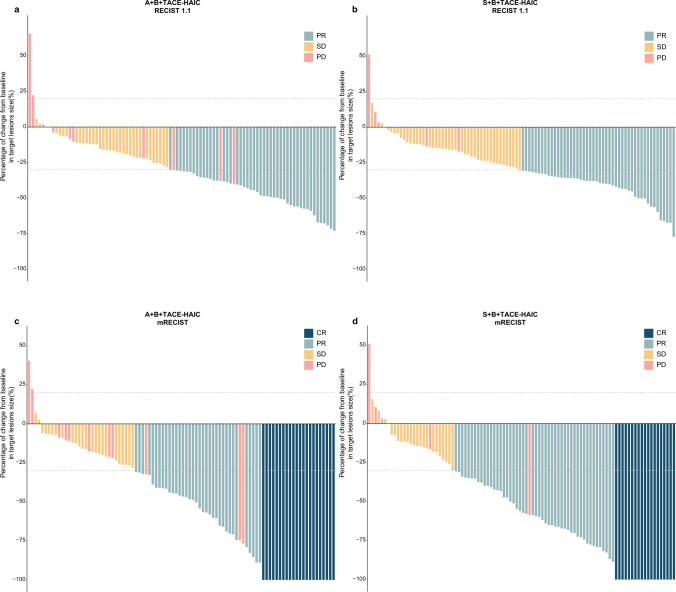
Percentage of changes from baseline in size of the intrahepatic target lesions

**Fig. 3:**
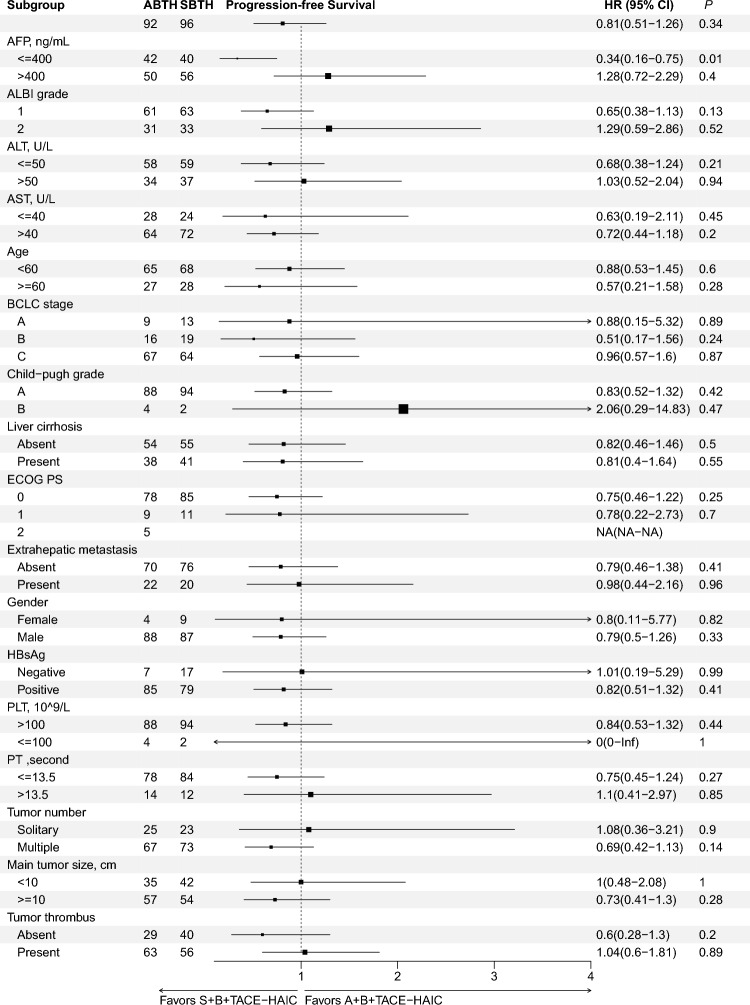
Forest plot for progression-free survival. ABTH, A+B+TACE-HAIC treatment; SBTH: S+B+TACE-HAIC treatment

As for intrahepatic target lesions, ORRs (65.2 vs. 72.9%, *P* = 0.325) and DCRs (97.8 vs. 100%, *P* = 0.459) were similar in the ABTH and SBTH groups, according to the mRECIST criteria. For overall response, ORRs (62.0 vs. 70.8%, *P* = 0.257) and DCRs (88.0 vs. 93.8%, *P* = 0.267) were similar in the ABTH and SBTH groups, according to the mRECIST criteria. By the time of analysis, PD was noted in 11 (12.0%) patients of the ABTH group and 6 (6.2%) of the SBTH group, with 4 (4.3%) and 5 (5.2%) deaths recorded in the study period, respectively. Detailed tumor responses are summarized in Table 2. Furthermore, after transitioning to resectable HCC, 18 (19.6%) patients in the ABTH group and 15 (15.6%) in the SBTH group were further administered curative hepatectomy, of whom 5 (5.4%) and 7 (7.3%) patients in the ABTH and SBTH groups achieved pathological CR (pCR), respectively. The waterfall plot depicts the best percentage changes from baseline in the size of intrahepatic target lesions according to the RECIST 1.1 (Fig. 2a, b) and mRECIST criteria (Fig. 2c, d). The forest plot analysis of factors associated with PFS is shown in Fig. 3. SBTH provided a PFS benefit in patients with AFP ≤ 400 ng/mL (HR = 0.35, 95% CI, 0.17–0.72,* P* = 0.005; Fig. 1c) compared to ABTH, but failed to confer a survival benefit in patients with other characteristics.

**Table 2:** Summary of best response according to RECIST1.1 and mRECIST

	RECIST 1.1 (%)			mRECIST (%)		
	ABTH (92)	SBTH (96)	*P*	ABTH (92)	SBTH (96)	*P*
*Overall*						
CR	0 (0.0)	0 (0.0)	NA	22 (23.9)	19 (19.8)	0.612
PR	47 (51.1)	48 (50.0)	0.998	35 (38.0)	49 (51.0)	0.1
SD	34 (37.0)	42 (43.8)	0.424	24 (26.1)	22 (22.9)	0.737
PD	11 (12.0)	6 (6.2)	0.267	11 (12.0)	6 (6.2)	0.267
ORR	47 (51.1)	48 (50.0)	0.998	57 (62.0)	68 (70.8)	0.257
DCR	81 (88.0)	90 (93.8)	0.267	81 (88.0)	90 (93.8)	0.267
*Intrahepatic*						
CR	0 (0.0)	0 (0.0)	NA	25 (27.2)	22 (22.9)	0.613
PR	50 (54.3)	51 (53.1)	0.983	35 (38.0)	48 (50.0)	0.133
SD	40 (43.5)	45 (46.9)	0.748	30 (32.6)	26 (27.1)	0.504
PD	2 ( 2.2)	0 (0.0)	0.459	2 (2.2)	0 (0.0)	0.459
ORR	50 (54.3)	51 (53.1)	0.983	60 (65.2)	70 (72.9)	0.325
DCR	90 (97.8)	96 (100.0)	0.459	90 (97.8)	96 (100.0)	0.459

CR, complete response; PR, partial response; SD, stable disease; PD, progressive disease; ORR, objective response rate; DCR, disease control rate; RECIST 1.1, Response Evaluation Criteria in Solid Tumors, version 1.1; mRECIST, modified RECIST

### Prognostic factors

The prognostic factors of survival are shown in Table 3. In multivariate analysis, variables statistically different in univariate analysis were included, as well as the mandatory inclusion of the treatment administered. The results showed AST > 40 U/L (*P* = 0.02; HR = 2.14, 95% CI, 1.13–4.04) and tumor number > 1 (HR = 2.65, 95% CI, 1.22–5.77, *P* = 0.014) were risk factors for PFS while extrahepatic metastasis was a risk factor for OS. However, systemic treatment was not an independent risk factor for PFS or OS.

**Table 3:** Univariate and multivariate analysis of risk factors for progression-free survival and overall survival

Characteristic	Univariate analysis			Multivariate analysis			Univariate analysis			Multivariate analysis		
	HR	95% CI	*P* value	HR	95% CI	*P* value	HR	95% CI	*P* value	HR	95% CI	*P* value
Treatment, ABTH/SBTH	0.81	0.51–1.26	0.344	0.76	0.47–1.22	0.261	1.19	0.32–4.43	0.796	1.30	0.34–4.94	0.698
Age, < / ≥ 60	0.63	0.36–1.09	0.101				0.78	0.16–3.75	0.754			
Gender, Female/Male	1.33	0.54–3.29	0.540				22.54	0.00-Inf	0.583			
ECOG PS, 0/1	2.22	1.17–4.22	0.015	1.19	0.47–3.04	0.710	7.19	1.79–28.91	0.005	4.15	0.98–17.59	0.054
ECOG PS, 0/2	0.00	0.00-Inf	0.995				0.00	0.00–Inf	0.998			
Child-pugh grade, A/B	4.75	2.05–11.01	< 0.001	2.75	0.79–9.60	0.112	7.58	0.93–61.64	0.058			
HBsAg, no/yes	1.66	0.76–3.61	0.202				0.53	0.11–2.58	0.435			
ALBI grade, 1/2	1.23	0.76–1.98	0.402				2.22	0.59–8.29	0.237			
ALT, U/L, ≤ / > 50	1.29	0.82–2.02	0.274				1.37	0.37–5.09	0.642			
AST, U/L, ≤ / > 40	2.79	1.51–5.17	0.001	2.14	1.13–4.04	0.020	39.13	0.12–Inf	0.215			
PLT, 10^9/L, > / ≤ 100	1.16	0.36–3.68	0.805				0.00	0.00–Inf	0.998			
PT,second, ≤ / > 13.5	1.49	0.86–2.59	0.160				1.69	0.35–8.14	0.514			
AFP, ng/mL, ≤ / > 400	1.37	0.87–2.17	0.178				3.00	0.62–14.43	0.171			
BCLC stage, A/B	1.64	0.59–4.61	0.346				1.00	0.00–Inf	> 0.999			
BCLC stage, A/C	2.71	1.08–6.75	0.033	0.89	0.28–2.84	0.894	41.91	0.00–Inf	0.431			
Tumor number, 1/> 1	2.39	1.31–4.36	0.004	2.65	1.22–5.77	0.014	35.94	0.09–Inf	0.243			
Tumor thrombus, no/yes	1.35	0.84–2.17	0.209				2.55	0.53–12.31	0.243			
Extrahepatic metastasis, no/yes	2.46	1.53–3.96	< 0.001	1.45	0.84–2.48	0.181	8.13	2.03–32.57	0.003	6.48	1.53–27.44	0.011
Liver cirrhosis, no/yes	1.06	0.67–1.68	0.797				0.43	0.09–2.07	0.293			
Main tumor size, cm, < / ≥ 10	1.34	0.84–2.13	0.214				6.53	0.82–52.23	0.077			

### Safety profiles

TRAEs are summarized in Supplementary Table S1. All TRAEs were manageable, and no toxicity-associated deaths occurred in the follow-up. The ABTH group had lower grade 1–2 neutropenia (23.9% vs. 39.6%, *P* = 0.032) and hemoglobin decrease (50.0% vs. 68.8%, *P* = 0.014) rates compared with the SBTH group, while no significant differences were observed in other TRAEs.

## Discussion

We found ORRs (62.0 vs. 70.8%, respectively) were similar in the ABTH and SBTH groups. Additionally, ABTH did not show a PFS advantage over SBTH, with mPFS times of 11.7 and 13.0 months, respectively. Most TRAEs were manageable, suggesting that both combination therapies are feasible options for uHCC. Due to the short follow-up period, the median survival of patients in both groups remained undetermined. Long-term efficacy in terms of OS remained undefined. Nevertheless, 1-year OS rates were 93.7% and 90.8% in the ABTH and SBTH groups, respectively. Furthermore, after conversion to resectable HCC, 18 (19.6%) patients in the ABTH group and 15 (15.6%) in the SBTH group were further administered curative hepatectomy, of whom 5 (5.4%) and 7 (7.3%) patients in the ABTH and SBTH groups achieved pCR, respectively. SBTH provided a PFS benefit in patients with AFP ≤ 400 ng/mL compared to ABTH, indicating that SBTH may be a favorable option in these patients.

This study revealed that ABTH/SBTH had significantly improved efficacy and prolonged survival in uHCC patients compared with the A+B/S+B regimens alone. A possible reason is the combination of systemic treatment and locoregional therapy. A retrospective analysis reported an ORR for a combined treatment involving HAIC, PD-1 inhibitors, and lenvatinib in advanced HCC of 40% [12]. The LetoHAIC study further detected an elevated ORR of 67.6% in advanced HCC patients treated with a regimen including lenvatinib, toripalimab, and HAIC [13]. Moreover, Zhou et al. presented an impressive ORR of 67.3% when combining atezolizumab, bevacizumab, and HAIC for the treatment of advanced HCC [14]. Jointly, these findings underscore the potential of combining local and systemic treatments to confer significant benefits to this patient population.

The efficacy of combination therapies with diverse mechanisms of action for HCC treatment has been underscored by the observed synergistic antitumor effects [15–19]. Primarily, FOLFOX-based HAIC enhances the antitumor properties of ICIs by promoting immunogenic cell death and ensures the concentration of the chemotherapeutic within the tumor, thus minimizing tumor burden [20, 21]. Previous studies further support this notion, revealing an appreciable surge in cytotoxic T lymphocyte rates post-TACE, suggesting an enhanced immunological response [20–22]. Additionally, bevacizumab has dual effects, thwarting immunosuppression and fostering T cell infiltration into the tumor microenvironment, thereby augmenting the antitumor activity of PD-1/PD-L1 antibodies [23, 24]. Besides, bevacizumab potentially normalizes tumoral vasculature, countering intratumoral hypoxia and enhancing the efficacy of the chemotherapeutic regimen FOLFOX [25].

The ABTH group had lower grade 1–2 neutropenia (23.9% vs. 39.6%, respectively) and hemoglobin decrease (50.0% vs. 68.8%, respectively) rates compared with the SBTH group, while no significant differences were observed in other TRAEs. The current findings corroborate the IMbrave150 and ORIENT-32 studies [6, 9], which identified similar irAEs related to the A+B/S+B regimens, including hypothyroidism. Notably, given the potent therapeutic effects of combined approaches, this study reported elevated incidence rates for AEs, specifically those related to TACE-HAIC, e.g., hepatic dysfunction and particular abdominal discomfort, surpassing rates documented in previous research [11, 25–28]. Importantly, no new or unexpected toxicities emerged in this study, confirming the feasibility and safety profile of this combination therapy.

Atezolizumab is a programmed death-ligand 1 (PD-L1) antibody that mainly blocks PD-L1 on tumor cells, while sintilimab is a PD-1 antibody blocking PD-1 on T cells. Because they target different immune checkpoints, ABTH and SBTH may show varying efficacy levels and treatment-related adverse events (TRAEs). A meta-analysis reported that anti-PD-1 therapy exhibited superior overall survival and progression-free survival versus anti-PD-L1 therapy across various tumor types, while no significant difference was found in safety profile [29]. Partially consistent with this study, no significant differences in TRAEs were observed between the ABTH and SBTH groups; however, both groups showed similar efficacy.

This study had certain limitations. First, this retrospective investigation was restricted to three medical centers in China, excluding international participation, and its relatively modest sample size may introduce bias. Therefore, larger studies involving multiple institutions and nations would bolster the validity of the current conclusions. Additionally, the retrospective nature of this study may have led to underestimations of both the dosing intensities and potential TRAEs. To address this, we are conducting a prospective clinical trial, ABILITY (NCT05751343), to further investigate the efficacy of atezolizumab plus bevacizumab combined with TACE-HAIC. More importantly, a head-to-head clinical trial to compare the efficacy and safety of atezolizumab plus bevacizumab and sintilimab plus bevacizumab both combined with TACE-HAIC is needed. Another limitation was the preliminary status of median OS data, attributed to short follow-up. As the study progresses, long-term survival data will be collected for a more detailed analysis.

## Conclusion

In conclusion, these findings suggested that either PD-L1 or PD-1 inhibitor plus bevacizumab combined with TACE-HAIC have similarly excellent therapeutic efficacy with manageable adverse events, representing promising treatment options for uHCC.

## Supplementary Information

Below is the link to the electronic supplementary material.

Patient selection flow (PDF 389 kb)

Supplementary file2 (DOC 52 kb)

## Data Availability

The datasets used and/or analyzed during the current study are available from the corresponding author on reasonable request.

## References

[1] Bruix J, et al. Systemic treatment of hepatocellular carcinoma: an EASL position paper. J Hepatol. 2021;75(4):960–74. 34256065 10.1016/j.jhep.2021.07.004

[2] Cheng A-L, et al. Efficacy and safety of sorafenib in patients in the Asia-Pacific region with advanced hepatocellular carcinoma: a phase III randomised, double-blind, placebo-controlled trial. Lancet Oncol. 2009;10(1):25–34. 19095497 10.1016/S1470-2045(08)70285-7

[3] El-Khoueiry AB, et al. Nivolumab in patients with advanced hepatocellular carcinoma (CheckMate 040): an open-label, non-comparative, phase 1/2 dose escalation and expansion trial. Lancet. 2017;389(10088):2492–502. 28434648 10.1016/S0140-6736(17)31046-2 PMC7539326

[4] Kudo M, et al. Lenvatinib versus sorafenib in first-line treatment of patients with unresectable hepatocellular carcinoma: a randomised phase 3 non-inferiority trial. Lancet. 2018;391(10126):1163–73. 29433850 10.1016/S0140-6736(18)30207-1

[5] Zhu AX, et al. Pembrolizumab in patients with advanced hepatocellular carcinoma previously treated with sorafenib (KEYNOTE-224): a non-randomised, open-label phase 2 trial. Lancet Oncol. 2018;19(7):940–52. 29875066 10.1016/S1470-2045(18)30351-6

[6] Finn RS, et al. Atezolizumab plus bevacizumab in unresectable hepatocellular carcinoma. N Engl J Med. 2020;382(20):1894–905. 32402160 10.1056/NEJMoa1915745

[7] Reig M, et al. BCLC strategy for prognosis prediction and treatment recommendation: the 2022 update. J Hepatol. 2022;76(3):681–93. 34801630 10.1016/j.jhep.2021.11.018 PMC8866082

[8] Llovet JM, et al. Lenvatinib plus pembrolizumab versus lenvatinib plus placebo for advanced hepatocellular carcinoma (LEAP-002): a randomised, double-blind, phase 3 trial. Lancet Oncol. 2023;24(12):1399–410. 38039993 10.1016/S1470-2045(23)00469-2

[9] Ren Z, et al. Sintilimab plus a bevacizumab biosimilar (IBI305) versus sorafenib in unresectable hepatocellular carcinoma (ORIENT-32): a randomised, open-label, phase 2–3 study. Lancet Oncol. 2021;22(7):977–90. 34143971 10.1016/S1470-2045(21)00252-7

[10] Li B, et al. Conversion to resectability using transarterial chemoembolization combined with hepatic arterial infusion chemotherapy for initially unresectable hepatocellular carcinoma. Ann Surg Open. 2021;2:057. 10.1097/AS9.0000000000000057 PMC10455427 37636551

[11] Yuan Y, et al. TACE-HAIC combined with targeted therapy and immunotherapy versus TACE alone for hepatocellular carcinoma with portal vein tumour thrombus: a propensity score matching study. Int J Surg. 2023;109(5):1222–30. 37026861 10.1097/JS9.0000000000000256 PMC10389515

[12] Mei J, et al. Hepatic arterial infusion chemotherapy combined with PD-1 inhibitors plus lenvatinib versus pd-1 inhibitors plus lenvatinib for advanced hepatocellular carcinoma. Front Oncol. 2021;11:618206. 33718175 10.3389/fonc.2021.618206 PMC7947809

[13] He M-K, et al. Lenvatinib, toripalimab, plus hepatic arterial infusion chemotherapyversuslenvatinib alone for advanced hepatocellular carcinoma. Ther Adv Med Oncol. 2021. 10.1177/17588359211002720. 33854567 10.1177/17588359211002720 PMC8010824

[14] Xin Y, et al. Efficacy and safety of atezolizumab plus bevacizumab combined with hepatic arterial infusion chemotherapy for advanced hepatocellular carcinoma. Front Immunol. 2022;13:929141. 35990634 10.3389/fimmu.2022.929141 PMC9388744

[15] Chung AS, Lee J, Ferrara N. Targeting the tumour vasculature: insights from physiological angiogenesis. Nat Rev Cancer. 2010;10(7):505–14. 20574450 10.1038/nrc2868

[16] Liu WM, et al. Pre-treatment with chemotherapy can enhance the antigenicity and immunogenicity of tumours by promoting adaptive immune responses. Br J Cancer. 2009;102(1):115–23. 19997099 10.1038/sj.bjc.6605465 PMC2813751

[17] Sonbol MB, et al. Systemic therapy and sequencing options in advanced hepatocellular carcinoma. JAMA Oncol. 2020;6(12):e204930. 33090186 10.1001/jamaoncol.2020.4930 PMC7582230

[18] Voron T, et al. VEGF-A modulates expression of inhibitory checkpoints on CD8^+^ T cells in tumors. J Exp Med. 2015;212(2):139–48. 25601652 10.1084/jem.20140559 PMC4322048

[19] Wu C-J, et al. Lenvatinib plus pembrolizumab for systemic therapy-naïve and -experienced unresectable hepatocellular carcinoma. Cancer Immunol Immunother. 2022;71(11):2631–43. 35347395 10.1007/s00262-022-03185-6 PMC9519717

[20] Llovet JM, et al. Locoregional therapies in the era of molecular and immune treatments for hepatocellular carcinoma. Nat Rev Gastroenterol Hepatol. 2021;18(5):293–313. 33510460 10.1038/s41575-020-00395-0

[21] Palmer DH, Malagari K, Kulik LM. Role of locoregional therapies in the wake of systemic therapy. J Hepatol. 2020;72(2):277–87. 31954492 10.1016/j.jhep.2019.09.023

[22] Brown ZJ, Hewitt DB, Pawlik TM. Combination therapies plus transarterial chemoembolization in hepatocellular carcinoma: a snapshot of clinical trial progress. Expert Opinion Investig Drugs. 2021;31(4):379–91. 10.1080/13543784.2022.2008355 34788184

[23] Deng H, et al. Dual vascular endothelial growth factor receptor and fibroblast growth factor receptor inhibition elicits antitumor immunity and enhances programmed cell death-1 checkpoint blockade in hepatocellular carcinoma. Liver Cancer. 2020;9(3):338–57. 32647635 10.1159/000505695 PMC7325120

[24] Hegde PS, Wallin JJ, Mancao C. Predictive markers of anti-VEGF and emerging role of angiogenesis inhibitors as immunotherapeutics. Semin Cancer Biol. 2018;52:117–24. 29229461 10.1016/j.semcancer.2017.12.002

[25] He M, et al. Sorafenib plus hepatic arterial infusion of oxaliplatin, fluorouracil, and leucovorin versus sorafenib alone for hepatocellular carcinoma with portal vein invasion. JAMA Oncol. 2019;5(7):953. 31070690 10.1001/jamaoncol.2019.0250 PMC6512278

[26] He M-K, et al. Hepatic artery infusion chemotherapy using mFOLFOX versus transarterial chemoembolization for massive unresectable hepatocellular carcinoma: a prospective non-randomized study. Chin J Cancer. 2017;36(1):83. 29061175 10.1186/s40880-017-0251-2 PMC5654007

[27] Kudo M, et al. Randomised, multicentre prospective trial of transarterial chemoembolisation (TACE) plus sorafenib as compared with TACE alone in patients with hepatocellular carcinoma: TACTICS trial. Gut. 2020;69(8):1492–501. 31801872 10.1136/gutjnl-2019-318934 PMC7398460

[28] Lyu N, et al. Hepatic arterial infusion of oxaliplatin plus fluorouracil/leucovorin versus sorafenib for advanced hepatocellular carcinoma. J Hepatol. 2018;69(1):60–9. 29471013 10.1016/j.jhep.2018.02.008

[29] Duan J, et al. Use of immunotherapy with programmed cell death 1 versus programmed cell death ligand 1 inhibitors in patients with cancer: a systematic review and meta-analysis. JAMA Oncol. 2020;6(3):375–84. 31876895 10.1001/jamaoncol.2019.5367 PMC6990765

