# Supplementary figures and images for: PD-L1 inhibitor versus PD-1 inhibitor plus bevacizumab with transvascular intervention in unresectable hepatocellular carcinoma
Source: Clin Exp Med. 2024 Jun 28;24(1):138. doi: 10.1007/s10238-024-01415-y (PMC11213731; doi:10.1007/s10238-024-01415-y)

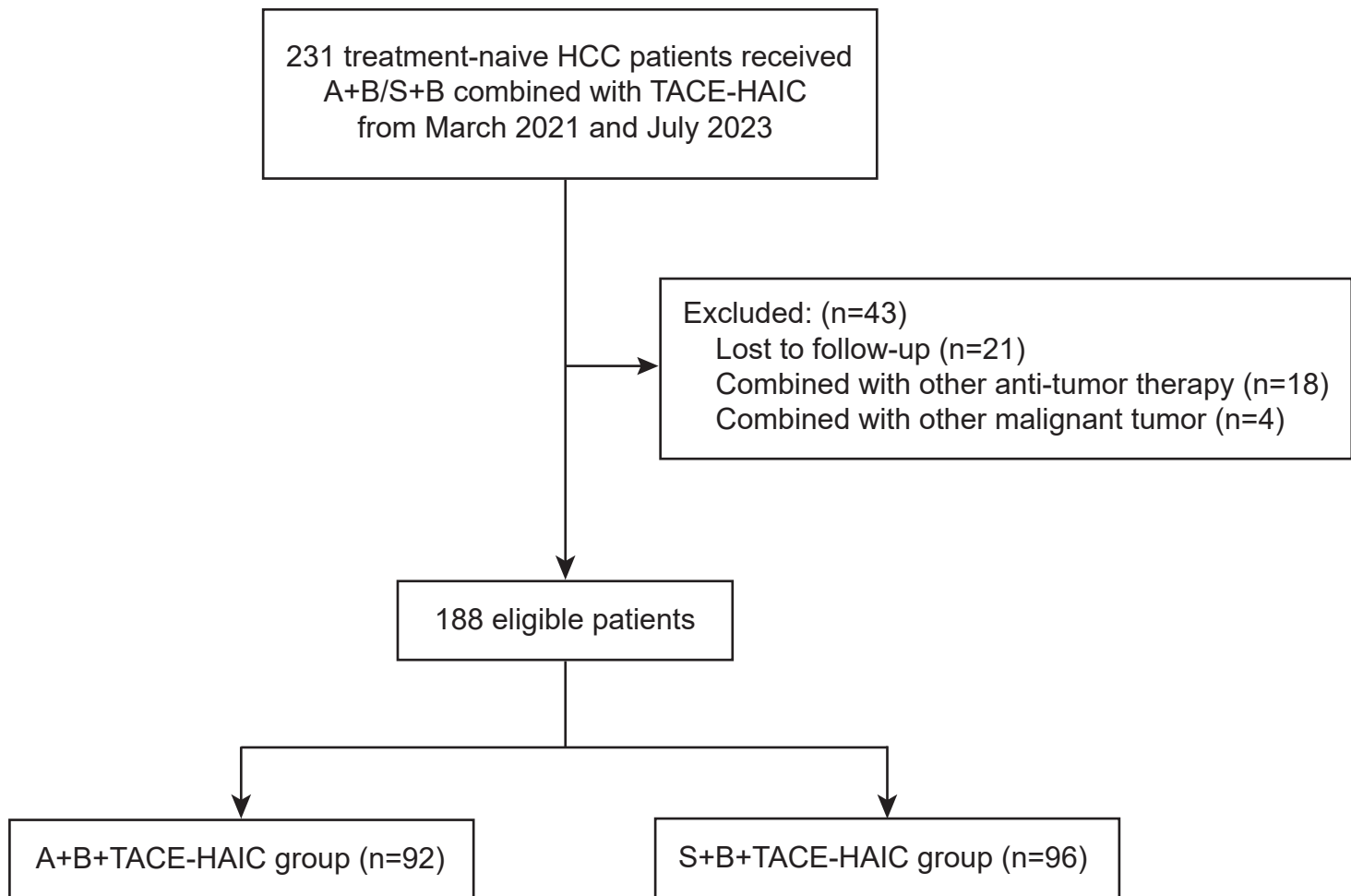

Supplement: Supplementary file 1 — Patient selection flow (PDF 389 kb) [file 10238_2024_1415_MOESM1_ESM.pdf]
