# Supplementary material for: PD-L1 inhibitor versus PD-1 inhibitor plus bevacizumab with transvascular intervention in unresectable hepatocellular carcinoma
Source: Clin Exp Med. 2024 Jun 28;24(1):138. doi: 10.1007/s10238-024-01415-y (PMC11213731; doi:10.1007/s10238-024-01415-y)
Supplement: Supplementary file 2 — Supplementary file2 (DOC 52 kb) [file 10238_2024_1415_MOESM2_ESM.doc]

Supplementary Table S1. Treatment-related adverse events for all grades.

| **Variable, n (%)** | **Any grade (%)** | |  | **Grade 3-4 (%)** | |  |
| --- | --- | --- | --- | --- | --- | --- |
|  | **ABTH (92)** | **SBTH (96)** | ***P*** | **ABTH (92)** | **SBTH (96)** | ***P*** |
| Nausea | 11 ( 12.0) | 15 ( 15.6) | 0.605 | 1 ( 1.1) | 2 ( 2.1) | 1 |
| Fatigue | 23 ( 25.0) | 30 ( 31.2) | 0.43 | 3 ( 3.3) | 4 ( 4.2) | 1 |
| Vomiting | 43 ( 46.7) | 50 ( 52.1) | 0.557 | 5 ( 5.4) | 7 ( 7.3) | 0.824 |
| Abdominal pain | 13 ( 14.1) | 17 ( 17.7) | 0.638 | 2 ( 2.2) | 2 ( 2.1) | 1 |
| Decreased appetite | 17 ( 18.5) | 23 ( 24.0) | 0.46 | 2 ( 2.2) | 3 ( 3.1) | 1 |
| Diarrhea | 22 ( 23.9) | 24 ( 25.0) | 0.997 | 2 ( 2.2) | 3 ( 3.1) | 1 |
| Pyrexia | 17 ( 18.5) | 23 ( 24.0) | 0.46 | 2 ( 2.2) | 3 ( 3.1) | 1 |
| Gastrointestinal hemorrhage | 3 ( 3.3) | 4 ( 4.2) | 1 | 0 (0.0) | 0 (0.0) | NA |
| Weight decrease | 13 ( 14.1) | 14 ( 14.6) | 1 | 0 (0.0) | 0 (0.0) | NA |
| Hypothyroidism | 15 ( 16.3) | 15 ( 15.6) | 1 | 0 (0.0) | 0 (0.0) | NA |
| Alanine aminotransferase increased | 61 ( 66.3) | 73 ( 76.0) | 0.189 | 11 ( 12.0) | 13 ( 13.5) | 0.915 |
| Aspertate aminotransferase increased | 87 ( 94.6) | 95 ( 99.0) | 0.194 | 27 ( 29.3) | 42 ( 43.8) | 0.058 |
| Albumin decreased | 89 ( 96.7) | 92 ( 95.8) | 1 | 1 ( 1.1) | 0 ( 0.0) | 0.983 |
| Total bilirubin increased | 57 ( 62.0) | 66 ( 68.8) | 0.409 | 7 ( 7.6) | 9 ( 9.4) | 0.863 |
| Leukopenia | 19 ( 20.7) | 33 ( 34.4) | 0.052 | 2 ( 2.2) | 5 ( 5.2) | 0.476 |
| Neutropenia | 22 ( 23.9) | 38 ( 39.6) | 0.032 | 6 ( 6.5) | 7 ( 7.3) | 1 |
| Hemoglobin decreased | 46 ( 50.0) | 66 ( 68.8) | 0.014 | 2 ( 2.2) | 2 ( 2.1) | 1 |
| Thrombocytopenia | 35 ( 38.0) | 34 ( 35.4) | 0.824 | 5 ( 5.4) | 7 ( 7.3) | 0.824 |
